# Lactate level and lactate clearance for acute kidney injury prediction among patients admitted with ST-segment elevation myocardial infarction: A retrospective cohort study

**DOI:** 10.3389/fcvm.2022.930202

**Published:** 2022-10-13

**Authors:** Xi Zhou, Yanlei He, Long Hu, Qianli Zhu, Qingcheng Lin, Xia Hong, Weijian Huang, Peiren Shan, Dongjie Liang

**Affiliations:** ^1^Department of Cardiac Care Unit, The First Affiliated Hospital of Wenzhou Medical University, Wenzhou, China; ^2^Department of Cardiology, The First Affiliated Hospital of Wenzhou Medical University, Wenzhou, China

**Keywords:** lactate, lactate clearance, acute kidney injury, myocardial infarction, prediction

## Abstract

**Background:**

Hyperlactatemia is a prognostic marker among patients with ST-segment elevation acute myocardial infarction (STEMI). However, the predictive value of lactate and the dynamic change associated with acute kidney injury (AKI) among patients with STEMI, remain poorly understood. We aimed to compare single lactate values at admission (Lac_adm_) and 12 h after admission (Lac_12h_) with lactate clearance (LC) 12 h after admission for AKI prediction in patients with STEMI.

**Methods:**

A total of 1,784 patients with STEMI were included. The study endpoint was AKI occurrence during hospitalization. The predictive value of lactate levels measured at admission and 12 h after admission and LC for AKI prediction was determined using multivariate logistic regression analyses and compared with receiver operator characteristic (ROC) curve analysis.

**Results:**

Overall, AKI was observed in 353 (19.8%) patients. In multivariate logistic regression analyses, Lac_adm_ ≥ 4.3 mmol/L (OR: 1.53; 95% CI: 1.01–2.30), Lac_12h_ ≥ 2.1 mmol/L (OR: 1.81; 95% CI: 1.36–2.42), and LC ≥ −7.5% (OR: 0.40; 95% CI: 0.30–0.53) were the independent predictive factors for AKI after adjusting for confounders. ROC curve analysis results revealed that Lac_12h_ (0.639; 95% CI: 0.616–0.661) exhibited a significantly higher area under the curve (AUC) than those of Lac_adm_ (0.551; 95% CI: 0.527–0.574) and LC (0.593; 95% CI: 0.570–0.616) in the prediction of AKI. LC (_△_AUC = 0.037, *p* < 0.001) and Lac_12h_ (_△_AUC = 0.017, *p* = 0.029) enhanced the discrimination capacity of Mehran Risk Score (MRS) for AKI among patients undergoing emergency coronary angiography.

**Conclusion:**

Lac_12h_ is more effective for AKI prediction among patients with STEMI than Lac_adm_ and LC. Furthermore, Lac_12h_ and LC enhance the prediction capacity of MRS for AKI among patients after emergency coronary angiography.

## Introduction

Acute kidney injury (AKI) is frequently observed in patients with ST-segment elevation myocardial infarction (STEMI) and is associated with worse short- and long-term cardiovascular outcomes and mortality (1–3). Identifying patients with STEMI who are at high risk of AKI is crucial for the implementation of personalized prophylactic treatment measures and mitigation strategies. Previous studies have revealed that contrast-induced nephropathy (CIN) is a main cause of AKI after primary percutaneous coronary intervention (PPCI) (3, 4). However, a recent study conducted by Schmucker et al. (5) revealed that the severity of STEMI and its hemodynamic alterations are predictors of AKI development rather than the amount or type of contrast media applied during PPCI.

Hyperlactatemia is a marker of tissue hypoperfusion and is recognized as an effective prognostic marker in critically ill patients, including patients with septic shock, cardiogenic shock (CS), cardiac arrest and acute coronary syndrome (ACS) (6–9), with mild hyperlactatemia being associated with increased mortality (10). Furthermore, Jansen et al. (11) performed a randomized controlled trial among patients with hyperlactatemia on intensive care unit (ICU) admission, and the results showed that early lactate clearance (LC)-guided therapy significantly reduced organ failure and hospital mortality. A recently published systematic review and meta-analysis identified high blood lactate levels as a predictor of AKI occurrence after cardiac arrest (12). Moreover, high lactate levels are independently associated with a high risk of post-operative renal dysfunction among patients undergoing cardiac surgery (13). However, the predictive value of lactate, especially lactate dynamics associated with AKI in the setting of acute myocardial infarction (AMI), remain poorly understood.

The present study aimed to investigate the predictive value of single arterial lactate indices at baseline and 12 h after admission and compare it to that of LC 12 h after admission for AKI prediction among patients with STEMI based on a large patient cohort, and to define cutoff values that may facilitate further risk stratification and therapy optimization. In addition, we aimed to assess whether incorporation of lactate indices into the Mehran Risk Score (MRS) may yield additional predictive information among patients with STEMI undergoing emergency coronary angiography.

## Materials and methods

### Study design and population

The present study was a retrospective, single-center study in which the medical records of 2,008 consecutive adult patients with a final diagnosis of STEMI who were admitted to the Coronary Care Unit (CCU) of the First Affiliated Hospital of Wenzhou Medical University between January 2014 and January 2019 were assessed. The exclusion criteria were patients with end-stage renal disease requiring chronic dialysis treatment, missing repeated serum creatinine measurements, and patients without admission lactate or repeated lactate measurements at 12 h. The study protocol was approved by the Ethics Committee of the First Affiliated Hospital of Wenzhou Medical University, and the need for written informed consent was waived.

### Data collection and definitions

Demographic and clinical data, including age, gender, previous medical history, initial vital signs, severity, dopamine use, laboratory data at the time of admission and in-hospital medications were obtained from electronic medical databases. Serum lactate levels were measured in arterial blood using commercially available ELISA kit. Lactate levels for patients at admission (Lac_adm_) and 12 ± 2 h (Lac_12h_) after admission were retrieved from the database. LC was defined as [(Lac_adm_ – Lac_12h_)/Lac_adm_] × 100. STEMI was defined based on the Third Universal Definition of Myocardial Infarction (14). Chronic kidney disease (CKD) was defined as an estimated glomerular filtration rate (eGFR) < 60 mL/min/1.73 m^2^ on admission based on the Chronic Kidney Disease Epidemiology Collaboration (CKD-EPI) equation (15). The MRS for patients who underwent emergency coronary angiography was calculated based on eight variables (hypotension, intra-aortic balloon pump [IABP], congestive heart failure, eGFR, diabetes, age > 75 years, anemia, and contrast volume) (16). In the present analysis, anemia was defined as baseline hemoglobin value of < 13 g/dL in men and < 12 g/dL in women. Congestive heart failure was defined as Killip classes II–IV. The outcome for the present study was the occurrence of AKI during hospitalization, which was defined as an absolute increase in creatinine level to ≥ 26.5 μmol/L within 48 h or an increase to ≥ 1.5-fold the baseline value, which was known or presumed to have occurred within the prior 7 days, according to the Kidney Disease: Improving Global Outcomes (KDIGO) guidelines (17). Urine output was not used as a criterion for AKI in the present study.

### Statistical analysis

Continuous variables were presented as means ± standard deviation, or medians with interquartile range (IQR) for variables with a skewed distribution. Categorical variables were presented as numbers and percentages. Group comparisons were performed using Student’s *t*-test or Mann–Whitney *U* test for continuous variables, and chi-squared (χ^2^) or Fisher’s exact test for categorical variables. Receiver operating characteristic (ROC) curves with their corresponding areas under the curves (AUCs) for Lac_adm_, Lac_12h_, and LC were calculated. The AUCs were compared using the method described by DeLong et al. (18). The best cutoff values for AKI prediction were obtained from ROC curve analysis using the Youden index. In addition, to evaluate whether each lactate index improved the predictive value of the MRS for AKI, we compared AUCs between MRS and MRS + lactate index. Univariate and multivariate logistic regression analyses were performed to determine the association between lactate index and AKI. Baseline variables with *p* < 0.10 in the univariate regression analysis [age, sex, hypertension, diabetes mellitus, current smoking status, current alcohol drinking status, history of previous stroke, CKD, emergency angiography, systolic blood pressure (SBP), heart rate, Killip classes II-IV, IABP use, dopamine use, diuretic, hemoglobin, glucose, Ln alanine transaminase (ALT), Ln B-type natriuretic peptide (BNP), Ln Hs-cTnI, and LVEF] were subjected to multivariate logistic regression analyses. The selected markers Lac_adm_, Lac_12h_, and LC were forced into the multivariate model. The results were presented as odds ratios (ORs) with 95% confidence intervals (CIs). Missing data on BNP (*n* = 4) were imputed as the mean values of the study population. Statistical analyses were performed using EmpowerStats^1^ and R^2^ (The R Foundation for Statistical Computing, Vienna, Austria). All *p*-values were two-sided, and *p* < 0.05 was regarded as statistically significant.

## Results

### Baseline characteristics of patients

**FIGURE 1:**
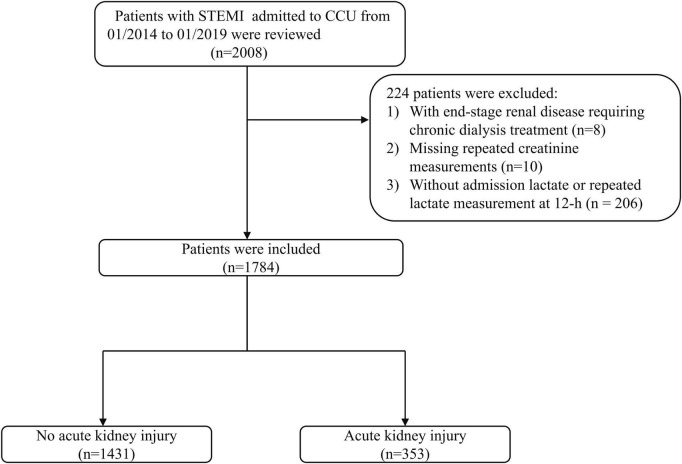
Study flowchart.

A total of 2,008 patients with STEMI who were admitted to CCU during the study period were reviewed. We excluded eight patients with end-stage renal disease requiring chronic dialysis treatment, 10 patients with missing repeated creatinine measurements, and 206 patients without admission lactate or repeated lactate measurements at 12 h, resulting in a cohort of 1,784 patients (Figure 1). The mean age was 64.3 ± 13.2 years, and 1,396 patients (78.3%) were men. Overall, AKI was observed in 353 (19.8%) patients. Table 1 shows the baseline clinical characteristics of patients with and without AKI. Patients with AKI were older and more likely to be female. Certain medical histories such as hypertension, diabetes mellitus, previous stroke, and CKD were frequently observed among patients with AKI. Patients with AKI were more likely to present with Killip classes II-IV, treated with IABP and dopamine, and less likely to undergo emergency angiography than patients without AKI. Patients with AKI were treated more frequently with diuretics during hospitalization and had higher heart rates, baseline glucose levels, creatinine levels, BNP, ALT, Hs-cTnI, and lower LVEF and hemoglobin levels than patients without AKI.

**TABLE 1:** Baseline characteristics of the study population.

n (%) or median (Interquartile range)	All (*n* = 1784)	No AKI (*n* = 1431)	AKI (*n* = 353)	*P*-value
**Demographic**				
Age, years	64.3 ± 13.2	63.1 ± 13.0	69.2 ± 12.7	< 0.001
Male	1,396 (78.3)	1,158 (80.9)	238 (67.4)	< 0.001
**Medical history**				
Hypertension	1,036 (58.1)	795 (55.6)	241 (68.3)	< 0.001
Diabetes mellitus	451 (25.3)	340 (23.8)	111 (31.4)	0.003
Hyperlipidemia	737 (41.3)	597 (41.7)	140 (39.7)	0.482
Current smoking	862 (48.3)	730 (51.0)	132 (37.4)	< 0.001
Current drinking	435 (24.4)	364 (25.4)	71 (20.1)	0.037
Prior MI	45 (2.5)	38 (2.7)	7 (2.0)	0.471
Prior PCI	90 (5.0)	67 (4.7)	23 (6.5)	0.159
Prior Stroke	114 (6.4)	75 (5.2)	39 (11.0)	< 0.001
CKD	288 (16.1)	174 (12.2)	114 (32.3)	< 0.001
**Presentation characteristics**				
Emergency angiography	1,622 (90.9)	1,311 (91.6)	311 (88.1)	0.040
PPCI	1,524 (85.4)	1,230 (86.0)	294 (83.3)	0.203
Contrast volume	90.0 ± 32.3	90.1 ± 32.3	89.3 ± 32.5	0.695
SBP, mmHg	123.4 ± 22.1	122.9 ± 21.5	125.3 ± 24.2	0.067
DBP, mmHg	75.1 ± 14.8	74.9 ± 14.6	75.8 ± 15.7	0.274
Heart rate, beats/min	82.8 ± 18.9	81.5 ± 18.3	88.0 ± 20.3	< 0.001
Killip class II-IV	467 (26.2)	301 (21.0)	166 (47.0)	< 0.001
Cardiogenic shock	168 (9.4)	97 (6.8)	71 (20.1)	< 0.001
IABP use	50 (2.8)	28 (2.0)	22 (6.2)	< 0.001
Dopamine use	123 (6.9)	80 (5.6)	43 (12.2)	< 0.001
**In-hospital medications**				
Aspirin	1,765 (98.9)	1,418 (99.1)	347 (98.3)	0.195
Clopidogrel or Ticagrelor	1,711 (95.9)	1,377 (96.2)	334 (94.6)	0.172
Statin	1,776 (99.6)	1,425 (99.6)	351 (99.4)	0.711
ACEI or ARB	973 (54.5)	785 (54.9)	188 (53.3)	0.589
Diuretic	682 (38.2)	479 (33.5)	203 (57.5)	< 0.001
**Laboratory data**				
Hemoglobin, g/L	130.0 ± 18.7	131.3 ± 17.9	124.5 ± 20.9	< 0.001
Glucose, mmol/L	8.9 ± 3.9	8.7 ± 3.6	9.9 ± 4.9	< 0.001
Creatinine, mmol/L	82.7 ± 66.5	78.7 ± 63.0	98.9 ± 77.0	< 0.001
ALT, U/L	55.0 (36.0–85.0)	54.0 (36.0–83.0)	58.0 (36.0–92.0)	0.008
BNP, ng/L	121.5 (45.0–373.0)	103.5 (38.0–284.2)	294.0 (96.0–842.0)	< 0.001
Hs-cTnI, ng/L	38.7 (10.6–50.0)	35.7 (9.8–50.0)	50.0 (15.1–50.0)	0.003
LVEF,%	47.9 ± 8.9	48.7 ± 8.7	43.9 ± 8.9	< 0.001
Lac_adm_	2.8 ± 1.8	2.7 ± 1.6	3.3 ± 2.5	< 0.001
Lac_12h_	2.3 ± 1.4	2.2 ± 1.0	3.0 ± 2.2	< 0.001
Lactate clearance	7.0 (−11.0–33.0)	10.0 (−6.0–34.0)	0.0 (−33.0–27.0)	< 0.001

MI, myocardial infarction; PPCI, primary percutaneous coronary intervention; CKD, chronic kidney disease; SBP, systolic blood pressure; DBP, diastolic blood pressure; IABP, intra-aortic balloon pump; ACEI, angiotensin-converting enzyme inhibitor, ARB, angiotensin receptor blocker; ALT, alanine transaminase; BNP, B-type natriuretic peptide; Hs-cTnI, high-sensitivity troponin I; LVEF, left ventricular ejection fraction. Continuous variables are presented as mean (standard deviation) for normally distributed variables or median [interquartile range (IQR)] for non-normally distributed variables, while categorical variables are presented as number (percentage).

### Association of lactate levels and clearance with acute kidney injury

**FIGURE 2:**
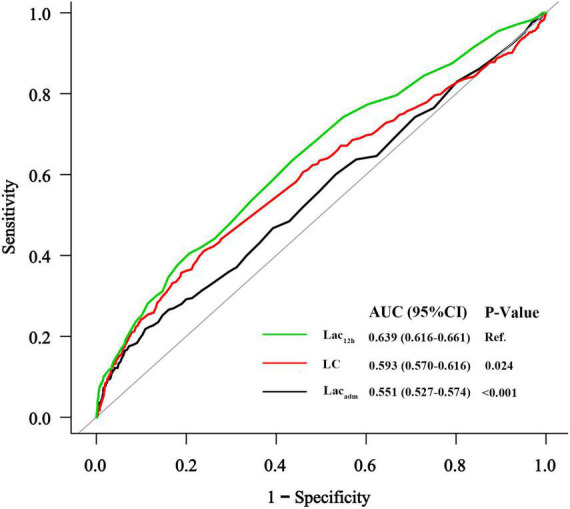
The receiver operating characteristic curves of lactate indices for AKI prediction.

In the study cohort, lactate levels (Lac_adm_ and Lac_12h_) and LC between patients with and without AKI were significantly different [patients without AKI vs. patients with AKI: Lac_adm_: 2.7 ± 1.6 mmol/L vs. 3.3 ± 2.5 mmol/L; *p* < 0.001; Lac_12h_: 2.2 ± 1.0 mmol/L vs. 3.0 ± 2.2 mmol/L; *p* < 0.001; LC: 10% (IQR: −6.0%–34%) vs. 0% (IQR: −33%–27%); *p* < 0.001] (Table 1). The best cutoff values for AKI prediction achieved by Youden index were arterial lactate levels ≥ 4.3 mmol/L (Lac_adm_), ≥ 2.1 mmol/L (Lac_12h_), and ≥ −7.5% (LC). Lac_adm_ [OR: 1.53; (95% CI: 1.01–2.30); *p* = 0.044], Lac_12h_ [OR: 1.81; (95% CI: 1.36–2.42); *p* < 0.001], and LC [OR: 0.40; (95% CI: 0.30–0.53); *p* < 0.001] were the independent predictive factors for AKI based on the previously mentioned cutoff values after adjusting for confounders in multivariate logistic regression analyses (Table 2). According to ROC curve analysis results, Lac_12h_ [0.639; (95% CI: 0.616–0.661)] had a significantly higher AUC than those of Lac_adm_ [0.551; (95% CI: 0.527–0.574); *p* < 0.001] and LC [0.593; (95% CI: 0.570–0.616); *p* = 0.024] in AKI prediction (Figure 2).

**TABLE 2:** Association of lactate variables with AKI.

	Univariate analyses			Multivariable analysis*		
	OR	95% CI	*P*-value	OR	95% CI	*P*-value
Lac_adm_ ≥ 4.3 mmol/l	2.28	1.69–3.09	< 0.001	1.53	1.01–2.30	0.044
Lac_12h_ ≥ 2.1 mmol/l	2.25	1.77–2.86	< 0.001	1.81	1.36–2.42	< 0.001
Lactate clearance ≥ −7.5%	0.45	0.35–0.58	< 0.001	0.40	0.30–0.53	< 0.001

*Beside the tested lactate variables, adjusted for age, sex, hypertension, diabetes mellitus, current smoking, current drinking, prior stroke, CKD, emergency angiography, SBP, heart rate, Killip class II-IV, IABP use, hemoglobin, glucose, Ln ALT, Ln BNP, Ln Hs-cTnI and LVEF.

### Incremental predictive value of lactate variables and Mehran risk scores

**FIGURE 3:**
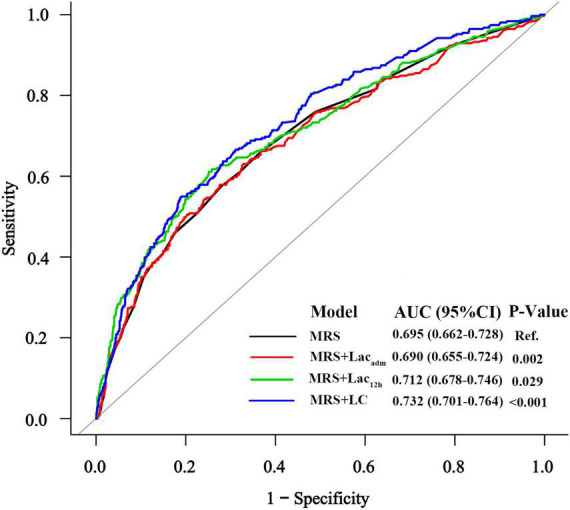
The receiver operating characteristic curves of MRS and MRS + lactate index for AKI prediction.

To determine whether each lactate index improved the predictive value of MRS for AKI in patients who underwent emergency coronary angiography (*n* = 1622), we compared AUCs between MRS and MRS + lactate index (Figure 3). The AUC for the prognostic model with MRS only was 0.695 (95% CI: 0.662–0.728), for the model with MRS + Lac_adm_ was 0.690 (95% CI: 0.655–0.724), for the model with MRS + Lac_12h_ was 0.712 (95% CI: 0.678–0.746), and for the model with MRS + LC was 0.732 (95% CI: 0.701–0.764). The AUCs of the MRS + Lac_12h_ (_△_AUC = 0.017, *p* = 0.029) and MRS + LC (_△_AUC = 0.037, *p* < 0.001) models were higher than that of the model with MRS only. AUC decreased significantly when Lac_adm_ was incorporated into the MRS only model (_△_AUC = −0.005, *p* = 0.002).

## Discussion

To the best of our knowledge, the present study is the first to assess the predictive value of single lactate indices and LC for AKI prediction among patients with STEMI. The major findings of the present study were: (i) Lac_adm_, Lac_12h_, and LC were the independent predictive factors for AKI among patients with STEMI; (ii) Lac_12h_ was superior to LC and Lac_adm_ in AKI prediction; (iii) Lac_12h_ and LC enhanced the capacity of MRS to predict AKI among patients after emergency coronary angiography. Thus, the clinical implication of this study is that dynamic monitoring of lactate levels facilitates early identification of high-risk patients for developing AKI among those with STEMI in routine clinical practice.

Previous studies have reported the prognostic role of lactate indices among patients with AMI. An observational study that included 1,176 patients with STEMI (19) showed that high lactate levels were independently correlated with 30-day mortality and an overall worse response to PCI. Similarly, a previous study performed by our research group demonstrated that Lac_adm_ was an independent predictor of 30-day and 180-day mortality among patients with ACS (9). In addition, a small pilot trial revealed that LC was a predictive factor of short and long-term mortality among patients with STEMI and CS (20). Fuernau et al. (7) recently conducted a sub-analysis of the IABP-SHOCK II (Intra-aortic Balloon Pump in Cardiogenic Shock II) trial and the corresponding registry to compare the prognostic impact of single lactate values at admission and 8 h after admission with those of LC during the early phase of infarct-related CS. In the study, arterial lactate was more effective in predicting patient death 8 h after admission than baseline lactate and LC. In contrast, few studies have investigated the relationship between arterial lactate and AKI occurrence among patients with STEMI. Considering the increased risk of adverse clinical outcomes among patients with STEMI who developed AKI, such investigations are relevant and essential. To date, only two studies with small sample sizes have investigated the relationship between lactate and AKI among patients with AMI. Yan et al. (21) analyzed 280 patients with AMI who underwent emergency PCI and demonstrated that high lactate levels at baseline were associated with an increased risk of contrast-induced AKI. A previous study that evaluated 227 patients with STEMI revealed that a pre-procedural arterial lactate level of ≥ 2.0 mmol/L was a predictive factor of CIN (22). However, both studies were limited by their small sample sizes and they only investigated the predictive value of baseline lactate levels. In contrast, the present study investigated the relationship between arterial lactate levels at two time points (baseline and 12 h after admission) and LC, as well as the risk of AKI in a large cohort of patients with STEMI. The results of the present study demonstrated that Lac_adm_, Lac_12h_, and LC were independent predictive factors of AKI among patients with STEMI after adjusting for baseline renal function and several other factors. Furthermore, Lac_12h_ was superior to Lac_adm_ and LC, as shown by ROC curve analysis.

Arterial lactate was recently introduced as a parameter in the CRATE score for AKI prediction among patients who underwent cardiac surgery (13). Moreover, Yang et al. (22) found that lactate level performance in predicting CIN among patients with STEMI following PPCI was similar to that of the MRS, a widely used and externally validated model (2, 23). However, data regarding the additional value of lactate parameters in MRS for AKI prediction among such patients is lacking. According to the results of the present study, Lac_12h_ and LC enhanced MRS capacity to predict AKI among patients with STEMI after emergency coronary angiography. Given the occurrence of AKI in STEMI patients is multifactorial, we chose AKI incidence as the endpoint and not contrast-induced AKI (CI-AKI) in the present study. As the MRS was a widely accepted score for CI-AKI prediction, whether lactate indexes enhanced the capacity of MRS to predict CI-AKI among patients after coronary angiography warrants further investigation. Nevertheless, further research should be conducted to develop a novel lactate-based risk score for predicting AKI among patients with STEMI.

The underlying pathophysiological mechanisms associated with AKI occurrence during hospitalization among patients with STEMI, including hemodynamically mediated kidney damage, exogenous factors (e.g., contrast media, angiotensin converting enzyme inhibitors, diuretics, etc.), neurohormonal activation, and immune mediated damage, are multifaceted and complex (24–26). Consequently, the relationship between lactate indices and AKI among patients with STEMI could be explained as follows. First, high lactate levels at admission in the case of STEMI suggest tissue hypoxia due to hemodynamic derangement and could therefore serve as a marker for inadequate renal perfusion (27). By contrast, subsequent arterial lactate levels and LC may reflect hemodynamic evolution among patients under therapy, such as emergency coronary revascularization. The observation could explain why arterial lactate levels at admission did not provide incremental predictive information for MRS in AKI, while Lac_12h_ and LC provided incremental predictive information as hemodynamic impairment in patients at presentation revealed by the Lac_adm_ had already been reflected by the parameters included in MRS, such as hypotension and heart failure. Second, hyperlactatemia could be indicative of response to stress, with increased sympathetic nervous system activation that plays a crucial role in AKI development (28). Notably, in the present study, patients with AKI demonstrated higher admission glucose values which always increase in response to stress. Adding pre-procedural glucose levels to MRS have been demonstrated to improve the predictive power of this score for CI-AKI in patients undergoing PCI (29). In addition, a study conducted by Jorge et al. found that abnormal combined lactate and glucose measurements can provide an early indication of renal dysfunction in critically ill patients (30). Furthermore, previous studies have reported that increased blood lactate levels were associated with high levels of endothelin-1, suggesting a more pronounced endothelin-mediated vasoconstriction at the periphery (31). Our findings indicate that lactate monitoring could be a valuable strategy for discriminating the risk of AKI among patients with STEMI; however, the underlying mechanisms remain indeterminate.

### Strengths and limitations

Strengths of this study comprise the large number of patients, the ability to analyze the dynamic changes of lactate, and completeness of the data. However, the present study had the following limitations. First, the study was a single-centered study; therefore, the results cannot be generalized to other populations with varying demographics. Second, the possibility of residual or unmeasured confounding that is typically associated with retrospective observational studies cannot be ruled out. For example, body mass index was unavailable for testing in our database. Third, the definition of AKI was based on the relative increase in creatinine levels when compared with baseline values, which may have already increased before hospital admission. In addition, urine output was not used as a criterion for AKI prediction in the present study. Therefore, AKI incidence could have been underestimated in our study, although the data reflect “real-world” clinical practice in an unselected cohort of patients with STEMI. Furthermore, the prevalence of CKD could be overestimated in this study as we defined it by a single eGFR measurement. Finally, we cannot exclude that lactate values measured at other time points may be better indicators when compared with values at baseline and 12 h after admission. Nevertheless, further research is required to determine whether arterial lactate could be a better indicator for AKI risk among patients with STEMI.

## Conclusion

The present study revealed that Lac_adm_, Lac_12h_, and LC were independently associated with a greater risk for AKI among patients with STEMI. Lac_12h_ was superior to Lac_adm_ and LC in AKI prediction. Furthermore, Lac_12h_ and LC enhanced the capacity of MRS to predict AKI among patients with STEMI after emergency coronary angiography. Therefore, lactate monitoring could be useful in predicting the risk of AKI among patients with STEMI.

## Data availability statement

The original contributions presented in this study are included in the article/supplementary material, further inquiries can be directed to the corresponding author/s.

## Ethics statement

The studies involving human participants were reviewed and approved by the Ethics Committee of the First Affiliated Hospital of Wenzhou Medical University. Written informed consent was not required for this study, in accordance with the local legislation and institutional requirements.

## Author contributions

XZ and DL contributed to the study design and drafted the manuscript. QZ, QL, and XH contributed to data collection. YH and LH analyzed the data and prepared figures. WH and PS revised the manuscript. DL provided the study supervision. All authors contributed to critical revision of the manuscript and approved its final version.

## Footnotes

1
http://www.empowerstats.com

2
https://www.r-project.org
